# Post-weaning diarrhea in pigs from a single Danish production herd was not associated with the pre-weaning fecal microbiota composition and diversity

**DOI:** 10.3389/fmicb.2023.1108197

**Published:** 2023-02-24

**Authors:** Martin Peter Rydal, Michela Gambino, Josue L. Castro-Mejia, Louise Ladefoged Poulsen, Claus Bøttcher Jørgensen, Jens Peter Nielsen

**Affiliations:** ^1^Department of Veterinary and Animal Sciences, Faculty of Health and Medical Sciences, University of Copenhagen, Frederiksberg, Denmark; ^2^Department of Food Science, Faculty of Science, University of Copenhagen, Frederiksberg, Denmark

**Keywords:** gastrointestinal microbiome, enterotoxigenic *Escherichia coli*, diarrhea, piglet, post-weaning, F18 fimbriae, high-throughput sequencing, genetic marker

## Abstract

**Introduction:**

The association between the porcine pre-weaning gut microbiota composition and diversity, and subsequent post-weaning diarrhea (PWD) susceptibility is currently being studied. In this longitudinal study, we examined the association between pre-weaning fecal microbiome composition and diversity, and PWD development in a Danish sow herd.

**Methods:**

Forty-five pigs were followed from birth until 7 days after weaning (post-natal day (PND) 33). At PND 33, the pigs were categorized as PWD cases or healthy controls based on fecal consistency. We compared their fecal microbiomes at PND 8, late lactation (PND 27) and 7 days post weaning (PND 33) using 16S rRNA V3 region high-throughput sequencing. At PND 27 and 33, we also weighed the pigs, assessed fecal shedding of hemolytic *Escherichia coli* by culture and characterized hemolytic isolates by ETEC virulence factors with PCR and by whole genome sequencing.

**Results:**

A total of 25 out of 45 pigs developed PWD and one Enterotoxigenic *E. coli* strain with F18:LT:EAST1 virotype was isolated from most pigs. At PND 33, we found differences in beta diversity between PWD and healthy pigs (*R^2^* = 0.027, *p* = 0.009) and that body weight was associated with both alpha and beta diversity. Pre-weaning fecal microbiome diversity did not differ between PWD and healthy pigs and we found no significant, differentially abundant bacteria between them.

**Conclusion:**

In the production herd under study, pre-weaning fecal microbiome diversity and composition were not useful indicators of PWD susceptibility.

## Introduction

The gut microbiota (GM) has an essential role in gut health (Wang et al., 2016). The composition of the microbial community is driven by host genetics (McKnite et al., 2012), diet (Frese et al., 2015), environmental conditions (Schmidt et al., 2011), and potentially physiological development (Leite et al., 2021). The rapid transition of diet in pig production, from milk as the primary nutrient source to solid feed at weaning, causes fast shifts in the GM composition and its enzymatic functional capacities (Frese et al., 2015). Taken together with the early time of weaning in pig production at three to four weeks of age (Jensen and Recén, 1989), and the stress associated with changes of environment, the weaning period is a vast challenge that can lead to gut microbiota dysbiosis; a state of gut microbial imbalance (Gresse et al., 2017) that may favor opportunistic pathogens. Consequently, post-weaning diarrhea (PWD) is very common in pig production and constitutes a serious problem in terms of animal welfare, financial costs and antibiotic use, where metaphylatic treatments are often used to control the disease (Rhouma et al., 2017). Post-weaning diarrhea is a multifactorial condition and enterotoxigenic *Escherichia coli* (ETEC) is the primary pathogen isolated from outbreaks (Dubreuil et al., 2016). Enterotoxigenic *E. coli* cause secretory diarrhea and are distinguished by their fimbriae type and the types of enterotoxins they produce. The fimbriae types most frequently associated with ETEC-related PWD are F18 and F4 (Frydendahl, 2002).

Recently, there has been increasing attention to the role of the GM in relation to PWD. Research shows that the early life environment affects microbiome composition and stimulates the maturation of the immune system (Mulder et al., 2009). Dysbiosis caused by unfavorable environmental conditions, such as limited or compromised exposure to microbes in the natural environment (Mulder et al., 2009; Schmidt et al., 2011), or by antibiotics early in life (Fouhse et al., 2019) may have long lasting consequences to host immune response (Mulder et al., 2009; Fouhse et al., 2019). This may consequently affect vulnerability to later infections as shown in experimentally infected mice exposed to early-life antibiotics (Roubaud-Baudron et al., 2019). Dou and colleagues found that pigs with PWD, under experimental facility conditions in France, differed in fecal microbiome diversity indices and composition from healthy pigs as early as 1 week after birth (Dou et al., 2017). Healthy pigs showed less evenness and higher abundance of taxa belonging to the families *Lactobacillaceae* and *Ruminocaceae* and other commensals. In a recent Czech herd study (Karasova et al., 2021), researchers investigated the role of the fecal microbiota in late lactation (3 days before weaning) in pigs with and without PWD. Healthy and PWD affected pigs differed in fecal microbiota composition, 3 days before weaning, although no extensive differences were found based on principal coordinate analysis. These findings indicate that certain GM communities might be associated with subsequent PWD development. However, more research is needed to elucidate if cues in the pre-weaning fecal microbiome such as diversity indices and abundance of certain taxa can reveal risk of PWD development. This may especially be relevant in a herd setting where management factors and antibiotic use can influence the GM.

In this study, we used 16S rRNA V3 high-throughput sequencing to investigate the fecal microbiome at early preweaning (post-natal day (PND) 8), at late lactation (PND 27) and at 7 days after weaning (PND 33). We determined diversity indices and microbiome composition and investigated their association with PWD development in pigs.

## Materials and methods

### Ethical statement

The study was ethically assessed and approved by the Animal Ethics Institutional Review Board at the Department of Veterinary and Animal Sciences, University of Copenhagen. Approval number: 2022-02-PNH-006A.

### Animals

A total of 45 female, Duroc x Landrace x Yorkshire pigs from 9 sows (5 pigs per sow) were included in the study. The herd of the study was part of the Danish SPF (Specific Pathogen Free) system, declared free from *Actinobacillus pleuropneumoniae* serotype 2, toxigenic *Pasteurella multocida*, *Brachyspira hyodysenteriae* and porcine reproductive and respiratory syndrome virus.

Female pigs, born on a single day, were weighed at birth. No crossfostering was performed in the pre-weaning period. Sow parity ranged from 2 to 5. The piglets were fed with milk formula through milk dispensers as a supplement to sow milk and were offered creep feed pre-weaning from PND 16. Pigs were weaned at PND 27 and received a standard weaner diet without added antimicrobials or medicinal zinc. All pigs had the same treatment history and received a single dose of amoxicillin trihydrate at post-natal day (PND) 1, Toltrazuril (anticoccidial) at PND 4, and metaphylatic lincomycin hydrochloride and spectinomycin sulfate (Linco-Spectin® Vet) treatment *via* drinking water during the entire study period after weaning. All 45 pigs were transported to and housed together at the nursery in a single pen, without additional pigs.

### Experimental design

The study was an observational case–control study with pig as the study unit. The study population was randomized in blocks by litter where five female pigs (birthweight higher than 1 kg) from each of 9 litters were selected out of available female pigs of the litter. Rectal swabs for fecal microbiome analysis were collected longitudinally from the pigs at PND 8, at late lactation (PND 27) and at 7 days after weaning (PND 33). In addition, at PND 27 and 33, fecal samples were collected rectally from the pigs for assessment of diarrhea and shedding of hemolytic *E. coli* and ETEC. Pigs were grouped based on fecal consistency at PND 33 into “PWD-cases” or “healthy controls” to compare their fecal microbiomes at the three time points.

### Fecal scoring and fecal dry matter content analysis

Feces were assessed using a visual fecal consistency score (1–4) (Pedersen and Toft, 2011) and diarrhea was defined as a fecal score greater than 2. Fecal dry matter content was determined by weighing feces before and after drying to constant weight, at 75°C for 18 h. Dried feces were kept in desiccators between weighings.

### Bacteriological culturing of fecal samples

Feces collected at PND 27 and 33 were streaked on blood agar (BA, 5% calf blood in blood agar base, Oxoid CM0055, ThermoFischer) in three dilution zones. The BA plates were incubated at 37°C overnight and semiquantitative assessment of hemolytic *E. coli* growth (%- hemolytic *E. coli* growth out of total growth, 0–100%) was performed. One haemolytic isolate per pig per timepoint was subjected to multiplex PCR to evaluate presence of the ETEC virulence factors F4, F18, STb, STa and LT with primers and protocol previously described (Zhang et al., 2007).

### Whole genome sequencing of *Escherichia coli* F18, LT isolates

Five randomly chosen *E. coli* F18, LT isolates were subjected to whole genome sequencing (WGS) for further characterization and to investigate if one or more strains were present. Extraction of DNA was performed on Maxwell® RSC instrument with Maxwell RSC culture cell’s DNA kit (Promega, Madison, USA) and WGS was performed at coverage of 30X, using 2×250 basepair V2 reagent kit with NGS-MiSeq (Illumina, University of Copenhagen, Denmark) platform, by service of Veterinary Clinical Microbiology at the Department of Veterinary and Animal Sciences, University of Copenhagen, Denmark. Raw sequencing reads were analyzed for virulence genes, serotypes and antibiotic resistance genes with the bioinformatics tools: VirulenceFinder (Version 2.0) (Joensen et al., 2014; Clausen et al., 2018; Tetzschner et al., 2020), SerotypeFinder (Version 2.0) (Joensen et al., 2015), and ResFinder (Version 4.1) (Zankari et al., 2017; Clausen et al., 2018; Bortolaia et al., 2020), hosted by the Center for Genomic Epidemiology (CGE) (www.genomicepidemiology.org).

### 16S rRNA V3 region high-throughput sequencing

Rectal swabs (Eswabs with Liquid Amies Medium, 490 CE.A, Copan Diagnostics Inc., Brescia, Italy) were stored at −80°C before DNA extraction. One rectal swab taken at PND 33 from a pig without PWD was missing before DNA extraction. Extraction of genomic DNA was carried out using Bead-Beat Micro AX Gravity kit (A&A Biotechnology, Gdynia, Poland) following the manufacturer’s instructions. DNA concentration was measured using Qubit® dsDNA HS Assay Kit (Life Technologies, CA, USA) with Varioskan Flash Multimode Reader (Thermo Fischer Scientific, MA, USA). Extracted DNA was diluted 1:10 with sterile water before PCR amplification of the V3 region of the 16S rRNA gene. The following primers were used: nxt_338_F: 5′-CCTACGGGWGGCAGCAG-3′ and nxt_518_R: 5′-ATTACCGCGGCTGCTGG-3′ (Integrated DNA Technologies; Leuven, Belgium). Reaction mix for PCR1 contained 5 μL of PCRBIO buffer, 0.5 μL primer mix (10 μM of each primer), 0.25 μL PCRBIO HiFi polymerase (PCR Biosystems Ltd., London, United Kingdom), 1 μL bovine serum albumin, 1 μL formamide, 12.25 μL nuclease-free water and 5 μL genomic DNA (approximately 1 ng/μL), per sample. The following thermal cycling was used: 95°C for 2 min, then 33 cycles of 95°C for 15 s, 55°C for 15 s, 72°C for 20 s, and 1 cycle of 72°C for 4 min. PCR1 products were verified and quality checked on 1.5% agarose gels. Products from PCR1 were then purified using AMPure XP beads (Beckman Coulter Genomic, CA, USA) with Biomek 4,000 automated laboratory workstation. Afterwards, barcoding (PCR2) of purified PCR products was performed. Reaction mix for PCR2 contained 5 μL PCRBIO buffer, 0.25 μL PCRBIO HiFi polymerase, 4 μL barcode primers (2 μL P5 and 2 μL P7 primers, Nextera Index Kit), 13.75 μL nuclease-free water and 2 μL PCR product, per sample. The cycling conditions for PCR2 were: 95°C for 1 min, then 13 cycles of 95°C for 15 s, 55°C for 15 s, 72°C for 15 s, and finally, 1 cycle of 72°C for 5 min. The barcoded PCR products were subjected to cleaning and bead-based normalization with AMPure XP beads. DNA concentration was measured once more and samples were pooled in equimolar concentrations. Finally, high throughput Illumina MiSeq (Illumina, CA, USA) sequencing was performed following the manufacturer’s instructions.

### Gut microbiota sequencing

Quality-control of reads, de-replicating, purging from chimeric reads and constructing zero-radius Operational Taxonomic Units (zOTU) was conducted with the UNOISE pipeline (Edgar, 2018) and taxonomically assigned with Sintax (Edgar, 2016). Taxonomical assignments were obtained using the EZtaxon for 16S rRNA gene database. Description of the workflow can be found in: https://github.com/jcame/Fastq_2_zOTUtable.

### FUT1 genotyping

All pigs were genotyped for the M307 mutation in FUT1. The M307 is a good marker for ETEC F18 resistant animals (Meijerink et al., 1997). Pig genomic DNA extraction was done from rectal swabs (Eswabs with Liquid Amies Medium, 490 CE.A, Copan Diagnostics Inc., Brescia, Italy). Genomic DNA was extracted from each swab using a crude DNA extraction method. Briefly, 100 μL medium was placed in a tube with 100 μL of NaOH solution (25 mM NaOH +2 mM EDTA). The sample was then heated at 100°C for 20 min. After centrifugation, the liquid phase was transferred and neutraliZed with 100 μL of Tris solution. The TaqMan SNP Genotyping assays were designed by Thermo Fisher Scientific (Waltham, Massachusetts, USA).

One μL of the crude DNA solution was used for TaqMan genotyping according to the manufacturer’s instructions. Allele calling was performed on a Mx3000P qPCR System (Agilent, Santa Clara, California, USA).

### Statistics

Statistical analysis was performed in R version 4.1.1 (R Core Team, 2022). *p*-values <0.05 were considered significant and < 0.10 a tendency.

Differences in fecal dry matter content and in shedding of hemolytic *E. coli* between diarrhea and non-diarrhea samples were evaluated with Wilcoxon rank sum test. Normal distribution of data was assessed using QQ-plots.

Association of litter and body weight with occurrence of PWD was evaluated with logistic regression.

For microbiome analysis and visualization, R packages vegan (Oksanen et al., 2022), phyloseq (McMurdie and Holmes, 2013), ANCOMBC (Mandal et al., 2015; Lin and Peddada, 2020), GUniFrac (Chen et al., 2022), lme4 (Bates et al., 2015), lmerTest (Kuznetsova et al., 2017), viridis (Garnier et al., 2021), tidyverse (Wickham et al., 2019), ggtext (Wilke and Wiernik, 2022), cowplot (Wilke, 2020), ggplot2 (Wickham, 2016), and RColorBrewer (Neuwirth, 2022) were used. Four samples were removed due to low number of reads (<10,000), resulting in n = 44, 43, 43 samples at PND 8, PND 27, and PND 33, respectively. All samples were rarefied to the same sequencing depth of 10,000 reads/sample.

Group differences in Canberra and binary Soerensen-Dice distance matrices between healthy and PWD pigs were assessed with PERMANOVA at 999 permutations. Model selection was based on forward selection using ordiR2step function of vegan. Group differences were assessed at each timepoint, with litter included in the model as strata. At PND 33, body weight at PND 33 was also included as a fixed effect in the model. Multivariate homogeneity of group dispersions were checked with betadisper function. Ordination plots were generated from the full model using capscale (distance-based redundancy analysis) function of vegan.

Significant group differences in Shannon and Simpson indices and number of observed zOTUs were evaluated with linear mixed model with litter included in the model as a random effect. At PND 33, body weight at PND 33 was also included as a fixed effect in the model.

Mean relative abundance between groups were calculated at genus level and visualized for each time point. Pearson’s correlation was calculated between relative abundance of *Prevotella* and body weight at PND 33.

Differential abundance analysis at each time point, was performed on amplicon counts at genus and zOTU level, using a taxon prevalence cut across samples of 10%, detecting structural zeros (using equation 1 in section 3.2 (Kaul et al., 2017)) and adjusting *p*-values with Holm–Bonferroni method. Data was analyzed with a univariable model (ANCOM-BC function) with PWD status as the explanatory variable and with a multivariable model (ANCOM function) where litter was added as random effect. At PND 33, body weight at PND 33 was also added to the model as fixed effect.

## Results

### Prevalence of diarrhea and risk factors for PWD

Just before weaning, at PND 27, only 1 out of 45 pigs had diarrhea. Four feces samples were missing at PND 27, as four pigs did not defecate at sample collection. Seven days after weaning, at PND 33, 25 out of the 45 pigs had diarrhea (Table 1). The majority of PWD samples were of watery consistency (16 out of 25 samples) indicative of severe diarrhea. Visual consistency scoring was confirmed by the significantly lower fecal dry matter content of PWD samples (*n* = 25) compared to non-diarrhea samples (*n* = 20) (Table 2, Wilcoxon test statistic (*W*) = 490, difference in medians = 12.33, 95% CI = 8.92;16.14, *p* < 0.0001).

**Table 1:** Prevalence of diarrhea and microbiological findings at late lactation (PND 27) and 7 days post weaning (PND 33).

	PND 27	PND 33
*E. coli* F18, LT [number of positive samples/samples total]	1/45	34/45
Pigs with diarrhea [*n* (%)]	1 (2%)	25 (55%)

**Table 2:** Clinical microbiology and fecal dry matter characteristics of feces at PND 33.

	Diarrhea	Non-diarrhea
Number of samples	25	20
Mean fecal dry matter ± SD [%]	11.3 ± 4.0	24.5 ± 6.6
Mean hemolytic *E. coli* shedding ± SD [% out of total growth]	76 ± 27	56 ± 33
*E. coli* F18, LT [number of positive samples/samples total]	18/25	16/20

Neither litter, birth weight (mean ± standard deviation (SD): 1.31 ± 0.18 kg and 1.38 ± 0.23 kg in PWD and healthy pigs, respectively) or body weight at PND 33 (mean ± SD: 8.8 ± 1.56 kg and 8.0 ± 1.6 kg in PWD and healthy pigs, respectively) had a significant association with PWD status in the pigs. A higher weaning weight was associated with slightly higher odds of PWD (mean weaning weight ± SD in PWD pigs were 8.66 ± 1.54 kg and 7.55 ± 1.69 kg in healthy, respectively, odds ratio = 1.54, 95% confidence interval (CI) = 1.05;2.4, *p* = 0.02).

### Microbiological findings

At PND 27, shedding of hemolytic *E. coli* was detected in a non-diarrheic sample from a single animal. Otherwise, no shedding of hemolytic *E. coli* was found before weaning. At PND 33, non-diarrhea samples tended to have less growth of hemolytic *E. coli* than the post-weaning diarrhea samples (Tables 2, *W* = 162, difference in medians = −20.0, 95% CI = −4.0;3.7, *p = 0.06*). Genes encoding for F18 and LT were detected by PCR in the hemolytic *E. coli* isolate from PND 27 and the majority of isolates at PND 33. No genes encoding for F4 were detected. Whole genome sequencing of five randomly chosen F18, LT positive isolates revealed the same virotype, thus confirming an ETEC F18 associated outbreak caused by a single strain. The herd strain was identified as O8:H17, F18ac, LT, EAST-1, with a WGS-predicted phenotype resistant to lincomycin, but not spectinomycin.

### Host genotype susceptibility to ETEC F18 using the FUT1-marker

Overall, 33 homozygous susceptible (SS), 11 heterozygous susceptible (RS) and 1 homozygous resistant (RR) pigs were identified by genotyping the FUT1-marker (Meijerink et al., 1997). The distribution of genotypes was similar between PWD cases and healthy controls (Table 3). Surprisingly, the single resistant pig was located in the PWD group which was confirmed by genotyping two independent samples from the same pig.

**Table 3:** FUT1 genotypes for ETEC F18 susceptibility between PWD status groups.

Groups	Post weaning diarrhea			Healthy		
Genotype profile	RR	RS	SS	RR	RS	SS
Number of pigs	1	8	16	0	3	17

### Microbiome analysis

#### Alpha diversity

**Figure 1:**
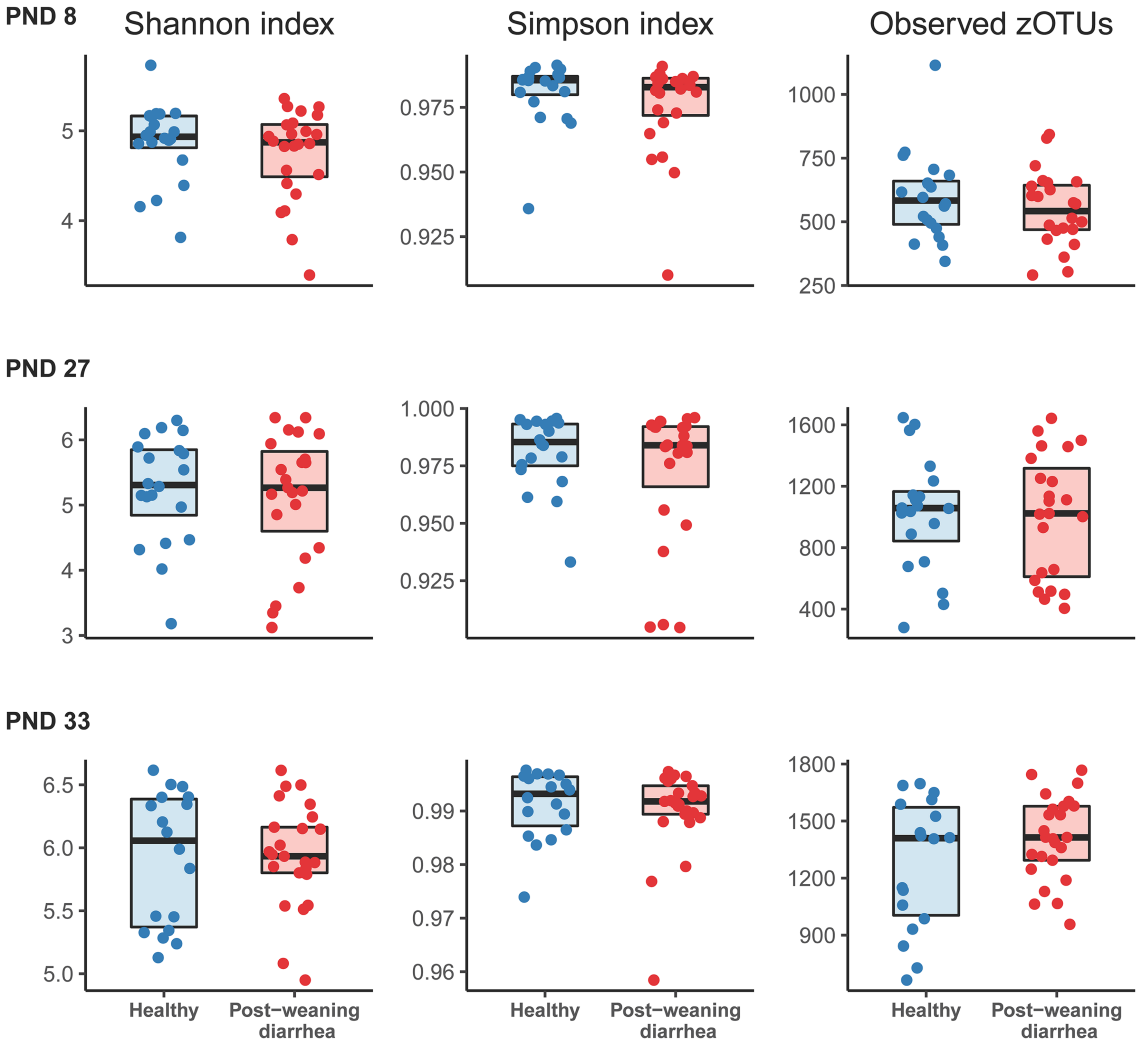
Comparison of alpha diversity indices based on post-weaning diarrhea (PWD) status. Groups were compared at post-natal day (PND) 8 (early lactation, healthy: *n* = 20 pigs, PWD: *n* = 24 pigs), at PND 27 (late lactation, healthy: *n* = 20 pigs, PWD: *n* = 23 pigs), and PND 33 (7 days after weaning, healthy: *n* = 18 pigs, PWD: *n* = 25 pigs). Shannon and Simpson indices and number of observed zOTUs were based on a rarefied zOTU table generated from 16S rRNA V3 high throughput amplicon sequencing. Graphs show boxplots with medians marked with bold line. Statistical differences between groups were assessed with linear mixed models. No significant group differences were found for PWD status at any time point. At PND 33, the covariable body weight at PND 33 was positively associated with increased Shannon diversity (effect size: 0.13, 95% CI: [0.068; 0.208], *p* = 0.0002), Simpson’s diversity (effect size: 0.001, 95% CI: [−0.00012; 0.002], *p* = 0.07), and number of observed zOTUs (effect size: 86.33, 95% CI: [46.27; 126.80], *p* = 0.00009).

In order to identify potential indicators for PWD early in pigs’ lives, we processed all fecal samples at PND 8, 27 and 33 with 16S rRNA V3 amplicon sequencing. In analysis of alpha diversity (Shannon and Simpson’s diversity index, and observed number of zOTUs), no statistically significant differences were found between the PWD group and healthy group at any timepoint (Figure 1). At PND 33, in the post weaning period, we found that the co-variable body weight was positively associated with increased alpha diversity. We did not find the same association between body weight and alpha diversity at PND 27 in the late lactation period.

#### Beta diversity

**Figure 2:**
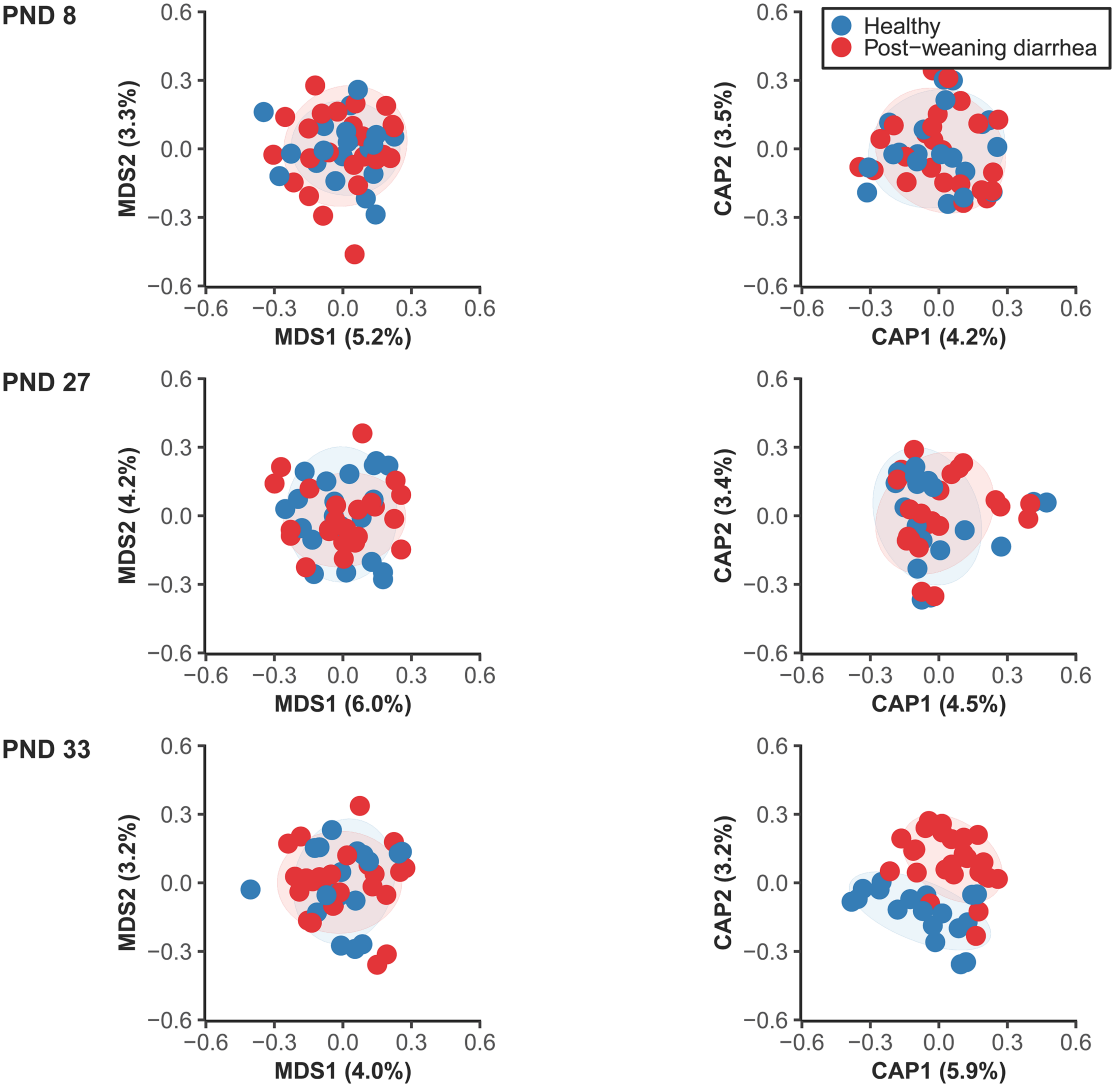
Unconstrained (left) and constrained ordination plots (right) of Canberra distance matrix of the fecal microbiome between healthy and post-weaning diarrhea (PWD) affected, at PND 8 (healthy: *n* = 20 pigs, PWD: *n* = 24 pigs), PND 27 (healthy: *n* = 20 pigs, PWD: *n* = 23 pigs), and PND 33 (healthy: *n* = 18 pigs, PWD: *n* = 25 pigs). Plots were created based on the rarefied zOTU table generated by 16S rRNA high throughput sequencing in the V3 region. Ellipses show 80% confidential areas assuming multivariate t-distribution. PERMANOVA was used to evaluate differences in distances at 999 permutations between the groups. Significant group difference in PWD status were found at PND 33: *R*^2^ = 0.027, *p* = 0.009. In addition, there was a significant effect of the covariable body weight at PND 33: *R*^2^ = 0.028, *p* = 0.003.

The analysis of beta diversity pre-weaning revealed no statistically significant differences at PND 8 (early lactation) nor at PND 27 (late lactation) based on PWD future status (Figure 2; Supplementary material 1). However at PND 33 (7 days after weaning), at the time of PWD occurrence, the fecal microbiotas of PWD pigs and healthy pigs had minor, but significant differences in their overall bacterial communities. In addition, we found that the co-variable body weight at PND 33 was also associated with differences in beta diversity at PND 33. In contrast, body weight was not associated with significant differences in beta diversity at PND 27 in the late lactation period.

There were no significant differences in dispersions between groups (Figure 2; Supplementary material 1) which confirmed that significant PERMANOVA results were due to location effects.

### Differential abundance analysis

**Figure 3:**
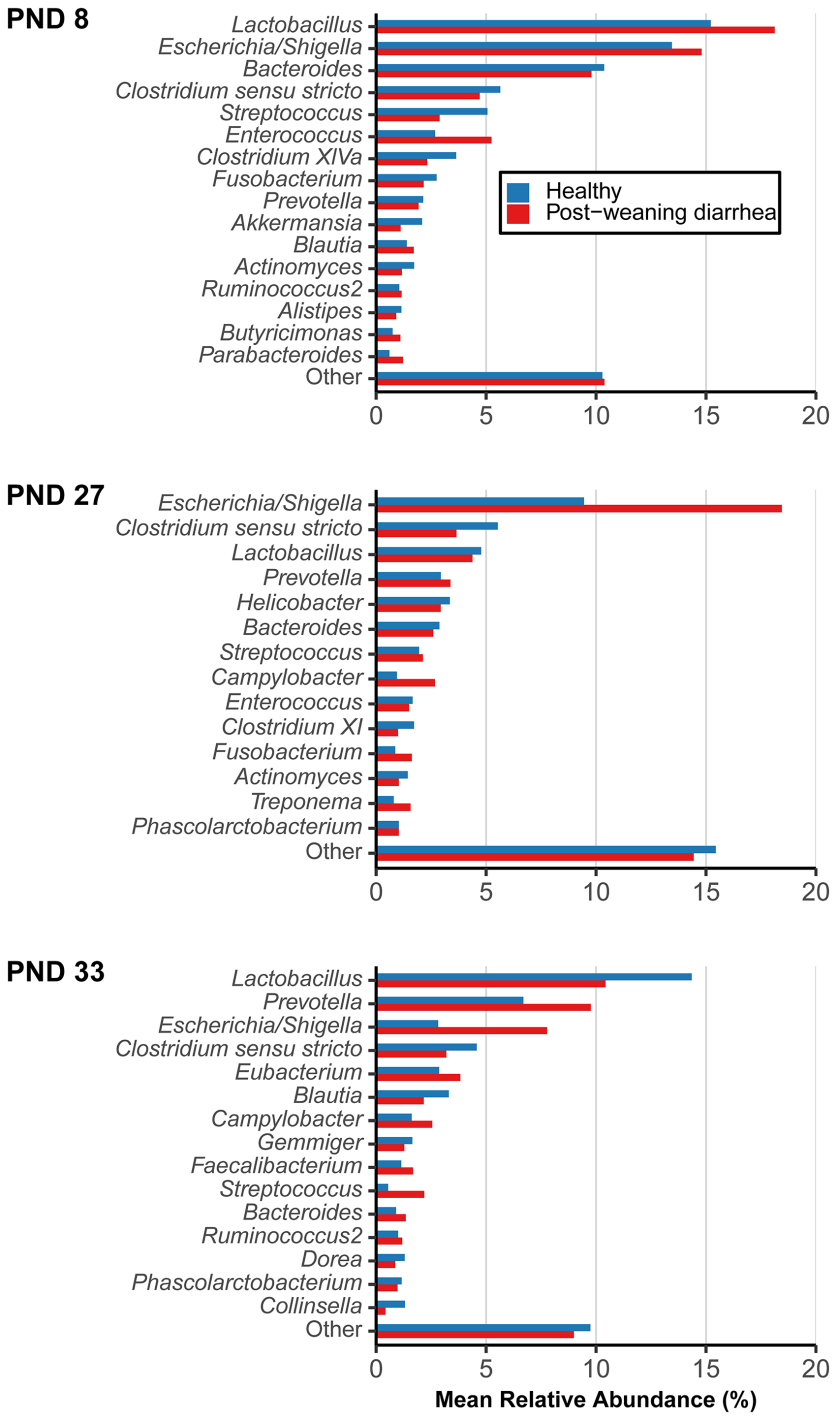
Mean relative abundance of most abundant genera, grouped by post-weaning diarrhea (PWD) status. Samples were collected longitudinally and the groups were compared at post-natal day (PND) 8 (early lactation, healthy: *n* = 20 pigs, PWD: *n* = 24 pigs), at PND 27 (late lactation, healthy: *n* = 20 pigs, PWD: *n* = 23 pigs), and PND 33 (7 days after weaning, healthy: *n* = 18 pigs, PWD: *n* = 25 pigs) which was the time of active PWD where pigs were divided into PWD cases and healthy controls for comparison. Genera representing <1% mean relative abundance were pooled into “Other.” Accumulated mean relative abundance of unassigned taxa (not shown) at genus level represented 20.00% (healthy) and 19.24% (PWD) at PND 8, 45.21% (healthy) and 37.62% (PWD) at PND 27, and 45.03% (healthy) and 41.36% (PWD) at PND 33.

Shift in the composition of bacterial communities were further investigated with differential abundance analysis. The most abundant bacterial genera and their mean relative abundance grouped by PWD status are illustrated in Figure 3. Four genera are of particular interest because of their dynamics in time: *Lactobacillus*, *Escherichia*/*Shigella*, *Prevotella*, *Campylobacter*. *Lactobacillus* and *Escherichia/Shigella* were the most dominant genera before weaning, while after weaning, the mean relative abundance of *Prevotella* increased in both groups (from 2.94% at PND 27 to 6.69% at PND 33 in healthy; from 3.37% at PND 27 to 9.76% at PND 33 in PWD pigs), making it the second most abundant genus post weaning. Interestingly, despite the ongoing ETEC associated PWD outbreak, the mean relative abundance of *Escherichia/Shigella* decreased at PND 33 both in healthy (from 9.45% at PND 27 to 2.82% at PND 33) and PWD pigs (from 18.44% at PND 27 to 7.77% at PND 33).

Agglomerating amplicon counts and analyzing at genus level did not reveal any differentially abundant genera based on PWD status at any individual timepoint. When we analyzed the data at a lower taxonomic level (zOTU level) we found differences in composition of microbial communities, but only as differences in presence/absence of rare taxa between groups.

At PND 8, 12 zOTUs were detected as structural zeroes (complete absence in one of the groups) (Supplementary material 2). The zOTUs did not belong to top abundant genera, except for two zOTU identified as *Prevotella* (mean relative abundance across taxa and samples 0.003%) that was found present only in the PWD group, and one zOTU identified as *Lactobacillus animalis* (mean relative abundance across taxa and samples 0.002%) that was only present in the healthy control.

At PND 27, a total of 17 zOTUs were detected as structural zeroes (Supplementary material 3). Among these were three zOTUs identified as *Lactobacillus* spp. (*Lactobacillus sanfranciscensis* and *Lactobacillus rapi,* mean relative abundance across taxa and samples at PND 27 of 0.013%) that were present only in healthy pigs, a genus that also had numerically higher mean relative abundance in healthy pigs (Figure 3 –PND 27).

At PND 33, a total of 99 zOTUs (98 of which were detected as structural zeroes) were differentially abundant between healthy and PWD pigs in the univariable analysis (Supplementary material 4). In the multivariable model, only the 98 zOTUs detected as structural zeroes remained differentially abundant between healthy and PWD pigs (Supplementary material 4). Four zOTUs identified as *Campylobacter hyointestinalis subsp. Lawsonii* (mean relative abundance across taxa and samples at PND 33 of 0.238%) were present only in PWD pigs. *Campylobacter* spp. was one of the top genera that had numerically higher mean relative abundance in PWD case pigs at PND 33 (Figure 3 –PND 33). Several zOTUs identified as *Prevotella* spp. were only detected in PWD pigs and this genus also had numerically higher mean relative abundance in the PWD affected pigs (Figure 3 –PND 33). Relative abundance of *Prevotella* at PND 33 increased with body weight at PND 33 (Supplementary material 5). Thus, questioning if the presence of *Prevotella* and numerically higher mean relative abundance in the PWD group was simply due to the higher average body weight in this group.

In summary, findings were limited to presence/absence of rare taxa between groups. Most of these differences were detected at PND 33 in the post-weaning period, and the least differences were found at PND 8 in the early lactation period.

## Discussion

In this study, we investigated whether PWD-case pigs and healthy pigs had different fecal microbiomes in the pre-weaning period. Such distinct patterns could be useful as early biomarkers for PWD susceptibility and give the scientific background for preventive measures. However, under our conditions, pigs that developed ETEC F18 associated PWD did not have distinguishable fecal microbiotas from their healthy counterparts before weaning.

This contrasts to the work of Dou and colleagues, who found that PWD pigs could be distinguished from healthy pigs that had lower evenness and higher abundance of *Ruminocacaceae*, *Prevotellaceae*, *Lachnospiraceae* and *Lactobacillaceae* already at PND 7 (Dou et al., 2017). An important difference from their study was that our study was performed in a herd setting with conditions close to reality at a farm. For example, as is common in pig farms to counteract umbilical cord infection, IM amoxicillin treatment was given to all pigs at PND 1. This may have perturbed the GM and made it harder to differentiate between PWD predisposed and healthy pigs 7 days after treatment. Transient perturbations of the GM has been observed in pigs receiving oral amoxicillin during the first 2 weeks of life (Fouhse et al., 2019). Immediate changes in the GM after IM amoxicillin has not been documented in pigs, but it was found to reduce bacterial community diversity in the colon 5 weeks after administration (Janczyk et al., 2007). However, in a recent herd study where pigs received creep feed with medicinal zinc pre-weaning and in-feed antibiotics (sulfamethoxazole and trimethoprim) after weaning, researchers still found several taxa in the fecal microbiome that were differentially abundant between their PWD-cases and healthy controls in the late pre-weaning period (Karasova et al., 2021). This suggests that it is possible to distinguish between PWD predisposed and healthy pigs regardless of previous and ongoing antimicrobial treatment. However, we found no obvious microbiome differences that could be used to identify PWD susceptible and resilient pigs in the pre-weaning period in our study. Although *Escherchia/Shigella* and *Lactobacillus* appeared more abundant based on the visualization of mean relative abundance at PND 27, we did not find these genera differentially abundant between future PWD cases and healthy controls when analyzing amplicon counts. At PND 27, a few low abundant zOTUs identified as *Lactobacillus* spp. were only present in healthy controls, but no significant differences in abundance were found in zOTUs related to this genus. Thereby, questioning if these structural zeros had any role the pre-weaning fecal microbiome of the healthy controls at PND 27. A possible explanation for the discrepancy between studies could also be that our study design and statistical analysis were more robust than previous studies.

In the present study, the sample size of pigs undergoing microbiome analysis was higher than in the two previous studies. In the study by Dou and colleagues, just 5 healthy and 5 diarrhoeic pigs were selected for bacterial 16S sequencing and compared. Karasova and colleagues, assessed slightly fewer pigs than us with 17 healthy and 17 pigs with PWD at each time point. The statistical analysis conducted in our study was also more conservative than previous studies. For example, we assessed confounding variables in our statistical analysis and accounted for the random effect of litter. Litter mates likely have bacterial compositions more similar to each other as their microbiota has been shaped in the same pen environment and they have the same mother (Grönlund et al., 2011). For the differential abundance analysis, we used ANCOM-BC and ANCOM which are designed for analysis of microbiome data and may be more reliable in determining differentially abundant taxa (Nearing et al., 2022). Finally, an advantage of our study was that we supported our 16S sequencing data with clinical microbiology and found that an ETEC F18ac strain was associated with PWD occurrence in the study. This meant that we could assess FUT1 genotype of the pigs to get an idea of host susceptibility to ETEC F18. We found that the distribution of FUT1 susceptible pigs were similar between groups and could therefore exclude the possibility that healthy pigs were merely resistant toward ETEC F18. We found a single pig with ETEC F18 diarrhea that was FUT1 homozygous resistant and we can only speculate on reasons for this finding. In theory, viruses or other factors could also have been present at the same time as ETEC F18 and be involved in occurrence of the diarrhea, but we did not investigate for presence of viruses in the study. Even more speculative, it could be that the marker is not the true causative mutation for ETEC F18 susceptibility, although this statement contradicts with the existing literature (Meijerink et al., 2000). This assertion would call for additional studies and cannot be concluded from this study. Dou and colleagues also genotyped their pigs to rule out that differences in host susceptibility to “*Enterobacteriaceae*-associated diarrhea” were involved in prevalence of PWD. However, they only used MUC13 genotyping, which is merely a marker for ETEC F4ac susceptibility (Ren et al., 2012), and did not investigate if ETEC F4ac strain(s) were actually present or associated with PWD occurrence in their study.

In relation to the types of bacteria that were most abundant at the different time points our results were quite comparable with previous literature. At PND 8, we found the most abundant bacteria were *Lactobacillus* spp., *Prevotella* spp., *Enterococcus* spp., *Bacteroides* spp. which is comparable to the OTUs that contributed most to group discrimination at PND 7 described by Dou and colleagues (Dou et al., 2017). At PND 27 (late lactation), we found family *Enterobacteriaceae*, *Lachnospiraceae, Ruminococcaeceae,* and *Lactobacillaeceae* (data not shown at family level, but described here for comparison) as the most abundant families, which were among the most abundant 3 days before weaning in the study by Karasova and colleagues (Karasova et al., 2021). We also observed a decrease of family *Enterobacteriaceae* and an increase in *Prevotellaceae* 7 days after weaning, similar to the observations by Karasova et al. at 4 days after weaning and to what has been described in literature (Frese et al., 2015; Mach et al., 2015).

As in previous studies, we did not collect fecal samples from the pigs every day in the 2 week period after weaning. Diarrhea occurring within this 2 week period is referred to as post-weaning diarrhea (Rhouma et al., 2017). Instead, we defined the pigs as PWD cases and healthy controls based on diarrhea occurrence at 7 days after weaning. This was the time where the farmer experienced problems with PWD in his herd and it was therefore decided to be the optimal sampling time. Diarrhea could also have occurred before or after our sampling, but it was not practically feasible at the herd to collect samples during the entire post weaning period and the same limitation apply to previous studies. Further, occurrence of diarrhea after weaning is not limited only to the first 2 weeks. However, we chose to focus on the period just after weaning, which is the period where classic, ETEC-associated post-weaning diarrhea occurs and where farmers experience most problems with diarrhea.

In the present study, the pigs with higher weaning weight had slightly higher odds of developing PWD. A possible explanation for this could be that the heavy weaners overate after weaning, which may result in increased risk for PWD (Hampson and Smith, 1986). We speculate that a higher solid feed intake of larger pigs results in more plant based substrate available and thereby promote and shape a gut microbiota with higher diversity of plant degrading bacteria (Tomova et al., 2019). This could also explain why we saw that increased body weight was associated with differences in beta diversity and a higher alpha diversity at PND 33. However, we did not measure feed intake after weaning which could have provided further insight in this. Another possible explanation for the higher odds of diarrhea in the heavier weaners could be that these pigs fought more to establish their position in the new hierarchy. This could result in additional stress and altered food and water intake (Coutellier et al., 2007) for the larger animals. It may be relevant for future similar studies to weigh the pigs at the time they are divided into disease cases and healthy controls to adjust for this potential confounder on microbiome diversity and composition.

In conclusion, the pre-weaning fecal microbiome did not contain biomarkers that could be used to identify PWD susceptible pigs in our herd. Instead, differences in the fecal microbiomes between PWD case pigs and healthy controls were first observed at 7 days after weaning. The lacking differences in the pre-weaning fecal microbiomes may be a result of similar poor gut microbiotas in both groups due to early life antibiotic treatments or be herd-specific. Alternatively, our findings may imply that the association between the pre-weaning fecal microbiome and PWD susceptibility has previously been overestimated perhaps due to small sample size and crude statistical analysis. More research is needed to assess if modulating the gut microbiota toward higher diversity and certain bacterial compositions in early-life pigs is a relevant strategy and investment for counteracting PWD.

## Data availability statement

The datasets generated/analyzed for this study can be found in its supplementary information files (Supplementary material 2–4 and 6–9). Supplementary material referred to in the article can be found in the article/Supplementary material. The data presented in the study are deposited in online repositories, with the following accession numbers: Whole genome sequences of the ETEC isolates as SRA data can be found at NCBI (BioProject: PRJNA905088) 16S amplicon sequence data as SRA data are available at NCBI (BioProject: PRJNA916113) SNPs can be found in the dbSNP data base: FUT1 (dbSNP:rs335979375).

## Ethics statement

The animal study was reviewed and approved by Animal Ethics Institutional Review Board at the Department of Veterinary and Animal Sciences, University of Copenhagen. Approval number: 2022-02-PNH-006A. Written informed consent was obtained from the owners for the participation of their animals in this study.

## Author contributions

MR designed the study, wrote the protocol, conducted the study, collected the data, did the statistical analysis, interpreted the data, and wrote the manuscript drafts. JN assisted in designing the study. MG, LP, and JN revised the protocol. MG and LP assisted and provided scientific input in microbiological assessment. JC-M provided scientific input for 16S rRNA sequencing, prepared the zOTU table and provided advice in statistical analysis of the microbiome data. CJ did FUT1 genotyping and assisted in interpretation of the genotyping results. All authors helped revising the manuscript drafts and approved the submitted version.

## Funding

This work was supported by the Innovation fund Denmark (IFD) (File number: 7076-00038B). The funding body did not play a role in the design, analysis, and reporting of the study.

## Conflict of interest

The authors declare that the research was conducted in the absence of any commercial or financial relationships that could be construed as a potential conflict of interest.

## Publisher’s note

All claims expressed in this article are solely those of the authors and do not necessarily represent those of their affiliated organizations, or those of the publisher, the editors and the reviewers. Any product that may be evaluated in this article, or claim that may be made by its manufacturer, is not guaranteed or endorsed by the publisher.

## Supplementary material

SUPPLEMENTARY MATERIAL 1 Unconstrained (left) and constrained ordination plots (right) of binary Soerensens dice distance matrix of the fecal microbiome between healthy and post weaning diarrhea (PWD) affected, at PND 8 (healthy: *n* = 20 pigs, PWD: *n* = 24 pigs), PND 27 (healthy: *n* = 20 pigs, PWD: *n* = 23 pigs), and PND 33 (healthy: *n* = 18 pigs, PWD: *n* = 25 pigs). Plots were created based on the rarefied zOTU table generated by Illumina sequencing in the V3 region. Ellipses show 80 % confidential areas assuming multivariate t-distribution. PERMANOVA was used to evaluate differences in distances at 999 permutations between the groups. Significant group difference in PWD status were found at PND 33: *R^2^* = 0.03, *p* = 0.012. Additionally, an effect of body weight at PND 33 was found: *R^2^* = 0.038, *p* = 0.002.

SUPPLEMENTARY MATERIAL 2 Results from differential abundance analysis at post-natal day 8.

SUPPLEMENTARY MATERIAL 3 Results from differential abundance analysis at post-natal day 27.

SUPPLEMENTARY MATERIAL 4 Results from differential abundance analysis at post-natal day 33.

SUPPLEMENTARY MATERIAL 5 Pearson’s correlation showed a positive correlation between relative abundance of genus *Prevotella* and body weight at PND 33: *r* = 0.32, 95 % confidence interval: [0.032;0.573], *p* = 0.03. *n* = 43 pigs.

SUPPLEMENTARY MATERIAL 6 Meta data for microbiome analysis.

SUPPLEMENTARY MATERIAL 7 zOTU table for microbiome analysis.

SUPPLEMENTARY MATERIAL 8 Taxonomy for microbiome analysis.

SUPPLEMENTARY MATERIAL 9 Data sheet for analysis of clinical data and microbiology.
