# Supplementary material for: Post-weaning diarrhea in pigs from a single Danish production herd was not associated with the pre-weaning fecal microbiota composition and diversity
Source: Front Microbiol. 2023 Feb 24;14:1108197. doi: 10.3389/fmicb.2023.1108197 (PMC10010570; doi:10.3389/fmicb.2023.1108197)
Supplement: SUPPLEMENTARY MATERIAL 1 — Unconstrained (left) and constrained ordination plots (right) of binary Soerensens dice distance matrix of the fecal microbiome between healthy and post weaning diarrhea (PWD) affected, at PND 8 (healthy: n = 20 pigs, PWD: n = 24 pigs), PND 27 (healthy: n = 20 pigs, PWD: n = 23 pigs), and PND 33 (healthy: n = 18 pigs, PWD: n = 25 pigs). Plots were created based on the rarefied zOTU table generated by Illumina sequencing in the V3 region. Ellipses show 80 % confidential areas assuming multivariate t-distribution. PERMANOVA was used to evaluate differences in distances at 999 permutations between the groups. Significant group difference in PWD status were found at PND 33: R2 = 0.03, p = 0.012. Additionally, an effect of body weight at PND 33 was found: R2 = 0.038, p = 0.002. [file Supplementary_material_1.docx]

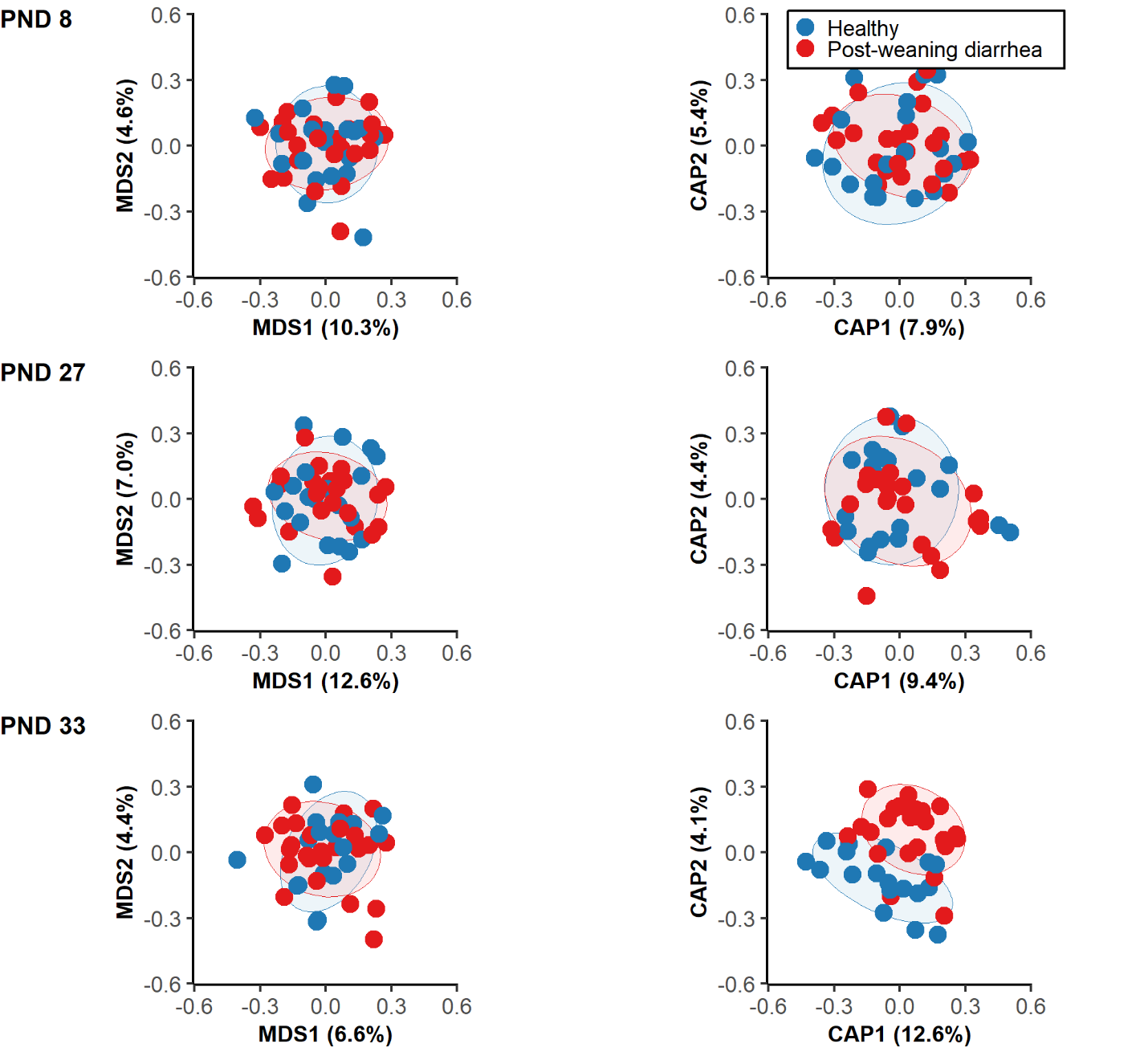


**Additional file 1.** Unconstrained (left) and constrained ordination plots (right) of binary Soerensens dice distance matrix of the fecal microbiome between healthy and post weaning diarrhea (PWD) affected, at PND 8 (healthy: n=20 pigs, PWD: n=24 pigs), PND 27 (healthy: n=20 pigs, PWD: n=23 pigs), and PND 33 (healthy: n=18 pigs, PWD: n=25 pigs). Plots were created based on the rarefied zOTU table generated by Illumina sequencing in the V3 region. Ellipses show 80 % confidential areas assuming multivariate t-distribution. PERMANOVA was used to evaluate differences in distances at 999 permutations between the groups. Significant group difference in PWD status were found at PND 33: R^2^=0.03, *p*=0.012. Additionally, an effect of body weight at PND 33 was found: R^2^=0.038, *p*=0.002.
