# Supplementary material for: Post-weaning diarrhea in pigs from a single Danish production herd was not associated with the pre-weaning fecal microbiota composition and diversity
Source: Front Microbiol. 2023 Feb 24;14:1108197. doi: 10.3389/fmicb.2023.1108197 (PMC10010570; doi:10.3389/fmicb.2023.1108197)
Supplement: SUPPLEMENTARY MATERIAL 5 — Pearson’s correlation showed a positive correlation between relative abundance of genus Prevotella and body weight at PND 33: r = 0.32, 95 % confidence interval: [0.032;0.573], p = 0.03. n = 43 pigs. [file Supplementary_material_5.docx]

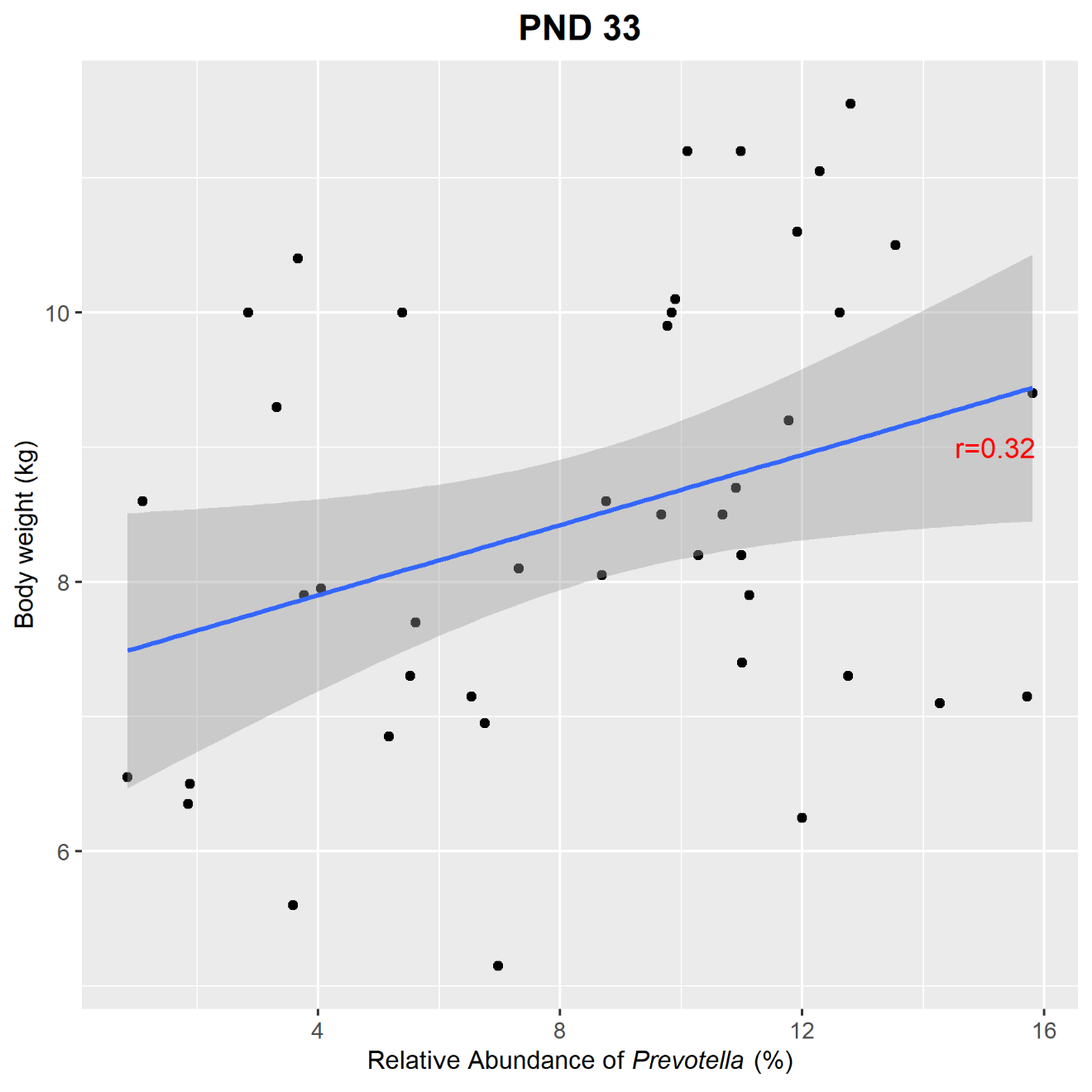


**Additional file 5.** Pearson’s correlation showed a positive correlation between relative abundance of genus *Prevotella* and body weight at PND 33: r=0.32, 95 % confidence interval: [0.032;0.573], *p*=0.03. n= 43 pigs.
